# Down-regulation of argininosuccinate synthetase is associated with cisplatin resistance in hepatocellular carcinoma cell lines: implications for PEGylated arginine deiminase combination therapy

**DOI:** 10.1186/1471-2407-14-621

**Published:** 2014-08-28

**Authors:** Jennifer A McAlpine, Hsin-Tze Lu, Katherine C Wu, Susan K Knowles, James A Thomson

**Affiliations:** Department of Biology, Polaris Pharmaceuticals, 9373 Towne Center Drive, Suite #150, San Diego, CA 92121 USA

**Keywords:** Arginine, Argininosuccinate synthetase, Arginine deiminase, Cisplatin, Hepatocellular carcinoma, Combination therapy

## Abstract

**Background:**

Many advanced human tumors, including hepatocellular carcinomas (HCC) are auxotrophic for arginine due to down-regulation of argininosuccinate synthetase (ASS1), the rate-limiting enzyme in arginine synthesis. The arginine-lowering agent PEGylated arginine deiminase (ADI-PEG 20) has shown efficacy as a monotherapy in clinical trials for treating arginine-auxotrophic tumors and is currently being evaluated in combination with cisplatin in other cancer types. Epigenetic silencing via methylation of the ASS1 promoter has been previously demonstrated in other cancer types, and a reciprocal relationship between ASS1 expression and cisplatin resistance has also been observed in ovarian cancer. However, the mechanism of ASS1 down-regulation, as well as the correlation with cisplatin resistance has not been explored in HCC. The present study investigates ADI-PEG 20 and cisplatin sensitivities in relation to ASS1 expression in HCC. In addition, we show how this biomarker is regulated by cisplatin alone and in combination with ADI-PEG 20.

**Methods:**

ASS1 protein expression in both untreated and drug treated human HCC cell lines was assessed by western blot. The correlation between ASS1 protein levels, ADI-PEG 20 sensitivity and cisplatin resistance in these cell lines was established using a luminescence-based cell viability assay. Epigenetic regulation of ASS1 was analyzed by bisulfite conversion and methylation-specific PCR.

**Results:**

A good correlation between absence of ASS1 protein expression, ASS1 promoter methylation, sensitivity to ADI-PEG 20 and resistance to cisplatin in HCC cell lines was observed. In addition, cisplatin treatment down-regulated ASS1 protein expression in select HCC cell lines. While, at clinically relevant concentrations, the combination of ADI-PEG 20 and cisplatin restored ASS1 protein levels in most of the cell lines studied.

**Conclusion:**

ASS1 silencing in HCC cell lines is associated with simultaneous cisplatin resistance and ADI-PEG 20 sensitivity which suggests a promising combination therapeutic strategy for the management of HCC.

## Background

Arginine, a semi-essential amino acid in humans, is critical for the growth of human cancers [1]. Tumoral down-regulation of the enzyme argininosuccinate synthetase (ASS1), the rate-limiting step in arginine synthesis, results in a critical dependence on extracellular arginine due to an inability to synthesize this amino acid from citrulline. Such a dependence on extracellular arginine is known as arginine auxotrophy. Many advanced human tumors more commonly associated with chemoresistance and poor clinical outcome, including hepatocellular carcinoma (HCC), melanoma, mesothelioma, pancreatic cancer, prostate cancer, renal cell carcinoma, sarcoma and small cell lung cancer, exhibit loss of ASS1 expression and are thus arginine auxotrophs [2–9]. Conversely, other tumor types such as colorectal, gastric and ovarian cancer tend to have higher expression of ASS1 [10, 11]. The mycoplasma-derived enzyme, arginine deiminase (ADI-PEG 20), PEGylated to enhance bioavailability and reduce immunogenicity, selectively degrades arginine, resulting in cell death in tumors lacking ASS1 [12]. Several phase I/II clinical trials of ADI-PEG 20 in patients with HCC and metastatic melanoma have shown promising indication of clinical benefit and low toxicity in patients with ASS1-deficient tumors [13–18]. A recently completed phase II trial of single-agent ADI-PEG 20 in ASS1-negative patients with mesothelioma also revealed encouraging efficacy results [19, 20].

The significance for ASS1 loss in cancer is currently unclear; however, several groups have revealed that reduced expression of ASS1 is a predictive biomarker for the development of metastasis and is associated with a worse clinical outcome [21–25]. Epigenetic silencing via methylation of the CpG islands within the ASS1 promoter accounts for loss of ASS1 expression in many solid tumors studied to date, including ovarian, malignant pleural mesothelioma, glioblastoma, myxofibrosarcoma and bladder, as well as in some lymphoid malignancies [4, 22–24, 26, 27]. Interestingly, the methylation status of ASS1 has been linked to platinum resistance in ovarian cancer [22]. Furthermore, it was found that patients treated with first line platinum/paclitaxel for ovarian cancer had a poor overall and disease-free survival if the tumor exhibited methylated ASS1 compared to unmethylated ASS1 [22, 28]. In addition, methylated ASS1 has been linked to increased proliferation and invasion of bladder cancer cells [24].

HCC is one of the most common cancers in the world, especially in Asia and Africa [29]. Cisplatin has been commonly used as a chemotherapeutic agent for HCC, but it has not satisfactorily improved the survival rate for patients with advanced HCC, either as a single agent or in combination, due to acquired or intrinsic drug resistance [30]. Intriguingly, drug resistance is an important contributor for treatment failure of ASS1-negative tumors by ADI-PEG 20, possibly due to re-expression of the once-silenced ASS1 that has been observed in melanoma cell lines [31–33]. To overcome this type of resistance, a second drug must be added to drive cell death. For example, it has been observed that the combination of ADI-PEG 20 and cisplatin can increase apoptosis in melanoma cell lines [34]. In addition, combined treatment of oxaliplatin and human arginase in HCC exhibited synergistic inhibiting effect on tumor growth [35], providing further support that a platinum and an arginine-deprivation agent would be a good combination in this cancer. ADI-PEG 20 is currently being utilized in several clinical trials, including a global phase III trial for HCC as a monotherapy, as well as in combination with cytotoxics such as cisplatin for the treatment of melanoma and ovarian cancer.

Previous work has shown that the sensitivity of HCC cell lines to ADI-PEG 20 is due to the absence of ASS1 [3]. However, the mechanism of ASS1 silencing, as well as the correlation with platinum resistance has not been explored in HCC. In addition, although ASS1 loss has been identified as a potential indicator of arginine auxotrophy in cancer, its regulation is complex and its use as a biomarker in combination therapy is unfamiliar. The current investigation was initiated to elucidate the relationship between ASS1 protein expression, ADI-PEG 20 sensitivity and cisplatin resistance, as well as to assess ASS1 regulation in response to cisplatin and in combination with ADI-PEG 20 in HCC. Utilizing several human HCC cell lines with varying amounts of ASS1, we report that ASS1 silencing confers sensitivity to ADI-PEG 20 and resistance to cisplatin. A good correlation is also observed between the methylation status of the ASS1 promoter, sensitivity to ADI-PEG 20 and resistance to cisplatin. In addition, cisplatin treatment down-regulates ASS1 protein expression in select HCC cell lines. Finally, the expression level of ASS1 during combination drug treatments with ADI-PEG 20 and cisplatin is cell line and concentration-dependent, but is predominantly dictated by ADI-PEG 20 at more clinically relevant concentrations. Taken together, our data indicate that ADI-PEG 20 and cisplatin will complement each other in a clinically relevant heterogeneous tumor, thus providing a rationale for combining these two drugs for the treatment of HCC.

## Methods

### Cell culture

The following human HCC cell lines were obtained from Dr. Yuh-Shan Jou at the Institute of Biomedical Sciences, Academia Sinica, Taipei, Taiwan: Sk-Hep1, Huh7, Tong, HCC36, Hep3B, Malhavu, PLC5 and Huh6. The human HCC cell lines HepG2, SNU398 and SNU182 were from American Type Culture Collection (ATCC, Manassas, VA). A2780 is an ovarian cancer cell line (cisplatin sensitive) derived from a patient prior to treatment and A2780CR is a cisplatin-resistant cell line that was developed by exposure of the parent A2780 cell line to increasing concentrations of cisplatin. Both A2780 and A2780CR cell lines were obtained from Sigma-Aldrich (St. Louis, MO). The following cells were grown in Dulbecco’s Modified Eagle Medium (DMEM) (Lonza, Allendale, NJ) containing 10% heat-inactivated fetal bovine serum (FBS; Life Technologies, Carlsbad, CA), 1% L-glutamine (Life Technologies) and 1% non-essential amino acids (NEAA, Life Technologies): Sk-Hep1, Huh7, Tong, HCC36, Hep3B, Malhavu, PLC5, Huh6 and HepG2. SNU398, SNU182 and the ovarian cell lines were maintained in RPMI 1640 (Lonza) with 10% heat-inactivated FBS and 1% L-glutamine. All cells were sub-cultured two times a week using trypsin/EDTA (Life Technologies) and were grown at 37°C in 5% CO_2_.

### Cell viability assay

Cell viability (IC_50_) values for ADI-PEG 20 and cisplatin (Sigma-Aldrich) were determined using the CellTiter-Glo (CTG) luminescent cell viability assay (Promega, Madison, WI). Cells (3,000-6,000 cells/well) were plated in 100 μL medium/well in 96-well black micro-clear plates (Greiner bio-one, Monroe, NC). Following overnight incubation at 37°C and 5% CO_2_, cells were exposed to a range of drug concentrations from a 50X plate (2 μL/well). Each concentration of drug was added to duplicate wells. After 72 h incubation, 25 μL/well of CTG reagent was added directly to the medium and the plates were shaken for 5 min, resulting in cell lysis and the generation of a luminescent signal proportional to the amount of ATP present. The luminescence values were read on a SpectraMax M3 microplate reader (Molecular Devices, Sunnyvale, CA) and converted to a percent cell viability that was calculated relative to the viability in corresponding matched DMSO-treated cells, which was designated as 100% viable. IC_50_ values (concentration of drug that results in 50% of luminescence signal compared with the DMSO-treated control) were obtained from nonlinear regression analysis of concentration-effect curves using GraphPad Prism version 6.0 software (San Diego, CA).

### Immunoblot analysis

Whole-cell extracts were made from 90% confluent cultures of all human cells. Cells were lysed in RIPA buffer (Sigma-Aldrich), with added protease inhibitor cocktail (Roche Molecular Systems, Pleasanton, CA) and PMSF (Sigma-Aldrich). Total lysate protein was quantified using a Coomassie Plus (Bradford) Protein Assay Reagent (Pierce, Rockford, IL). Cell extracts (20 μg) were run on NuPage 4-12% Bis-Tris Gels (Life Technologies) and then transferred to PVDF membranes (Life Technologies). The membranes were blocked in TBST buffer (Tris-HCL, 0.1% Tween) containing 5% Blotting-Grade Blocker (Bio-Rad, Hercules, CA) for 2 h at room temperature and then probed using a mouse monoclonal antibody to ASS1 (Polaris Pharmaceuticals, in-house) at a dilution of 1:500. GAPDH was used as a loading control for each western blot, so the membranes were cut and also probed with a rabbit polyclonal antibody to GAPDH (Millipore, Billerica, MA) at a dilution of 1:10,000. The blots were incubated with both primary antibodies overnight at 4°C on a rocker. After washing with TBST buffer, the membranes were incubated with secondary antibodies: goat anti-mouse for ASS1 (Santa Cruz Biotechnology, Dallas, TX) (1:10,000) and goat anti-rabbit for GAPDH (Santa Cruz Biotechnology) (1:60,000) and incubated at room temperature for 1 h. The secondary antibodies were detected using either the SuperSignal West Pico (GAPDH) or Femto (ASS1) Chemiluminescent Substrate (Pierce) and blots were read on a Bio-Rad ChemiDox XRS + System. ASS1 and GAPDH levels were quantified using Image Lab Software (Bio-Rad, Hercules, CA).

For ASS1 protein determination after cisplatin treatments or for ADI-PEG 20 and cisplatin combination analysis, the same procedure was used with the following modifications. Cells were plated in two identical 96-well plates: one for cell viability and/or normalization for cell numbers between wells (luminescence assay; see Methods above) and one for lysis (ASS1 detection). After 72 h drug treatments, lysates were made and probed for protein analysis. For ASS1 and GAPDH detection, media was removed and each well of the microplate was washed with 100 μL of PBS buffer (Gibco by Life Technologies). NuPage LDS sample buffer (30 μL of 1x sample buffer, Life Technologies) containing 50 mM DTT was then added to each well and the plate was wrapped in parafilm and frozen at -80°C for at least one hour to ensure lysis. After lysis, the samples in each well were spun and then used for immunoblot analysis. To account for the different number of viable cells in each well of the microplate, samples were normalized using the relative luminescence values for each corresponding well of the identical microplate.

### Bisulfite modification and methylation-specific PCR

The EZ DNA Methylation-Direct Kit (Zymo Research Corporation, Irvine, CA) was used to perform complete DNA bisulfite conversion directly from the human cell lines. This process converts unmethylated cytosine residues to uracil while methylated cytosine residues remain unchanged. In general, 10,000-40,000 cells (~60-250 ng genomic DNA) were used as starting material for each cell line. Methylation-specific PCR (MSP) of a 188 bp fragment located between 300 and 500 bp downstream of the transcription start site (TSS) was then performed to determine the methylation status of the ASS1 promoter. Bisulfite-modified DNA (150 ng) was used as a template for PCR reactions with primers specific for methylated (M) or unmethylated (UM) sequences. Primer sequences are: (1) M forward: 5′-TTTTTTTCGTTGTTTATTTTTTAGTC-3′; (2) M reverse: 5′-CTAAAATCCGATACCAAACGTT-3′; (3) UM forward: 5′-TTTT TGTTGTTTATTTTTTAGTTGA-3′ and (4) UM reverse: 5′-AACCTAAAATCCAATACCAAACATT-3′. Primers were purchased from IDT Technologies (San Diego, CA). PCR conditions for the methylated primers were as follows: 8 cycles of 95°C for 2 min, 54.8°C for 30 sec and 72°C for 30 sec were followed by 32 cycles of 95°C for 30 sec, 54.8°C for 30 sec and 72°C for 30 sec, then a final extension at 72°C for 10 min. The PCR conditions for the unmethylated primers were identical, except the annealing temperature was 48°C instead of 54.8°C. The HotStarTaq d-Tect polymerase (EpiTect MSP kit; Qiagen, Valencia, CA) was used for the PCR reactions. The PCR samples were run on 2% pre-cast agarose E-gels (Life Technologies) containing a fluorescent stain for visualization. The human methylated and non-methylated DNA set (Zymo Research) were used as negative and positive controls for bisulfite conversion efficiency, and MSP was similarly performed using a set of control primers designed to amplify non-methylated, methylated and mixed methylation copies of the death-associated protein kinase 1 gene (DAPK1), with an expected size of 274 bp.

### Statistical analysis

GraphPad Prism version 6.0 was used to test results for statistical significance. Differences in ASS1 levels between groups were analyzed using an unpaired two-tailed *t*-test. A p value < 0.05 was set as a level of statistical significance. In determining statistical significance for ASS1 protein levels after cisplatin treatments or for ADI-PEG 20 and cisplatin combination analysis, each drug concentration was compared to the untreated, or zero drug, sample to attain a p value for that particular drug concentration.

## Results

### ASS1 deficiency confers sensitivity to ADI-PEG 20 and resistance to cisplatin

**Figure 1:**
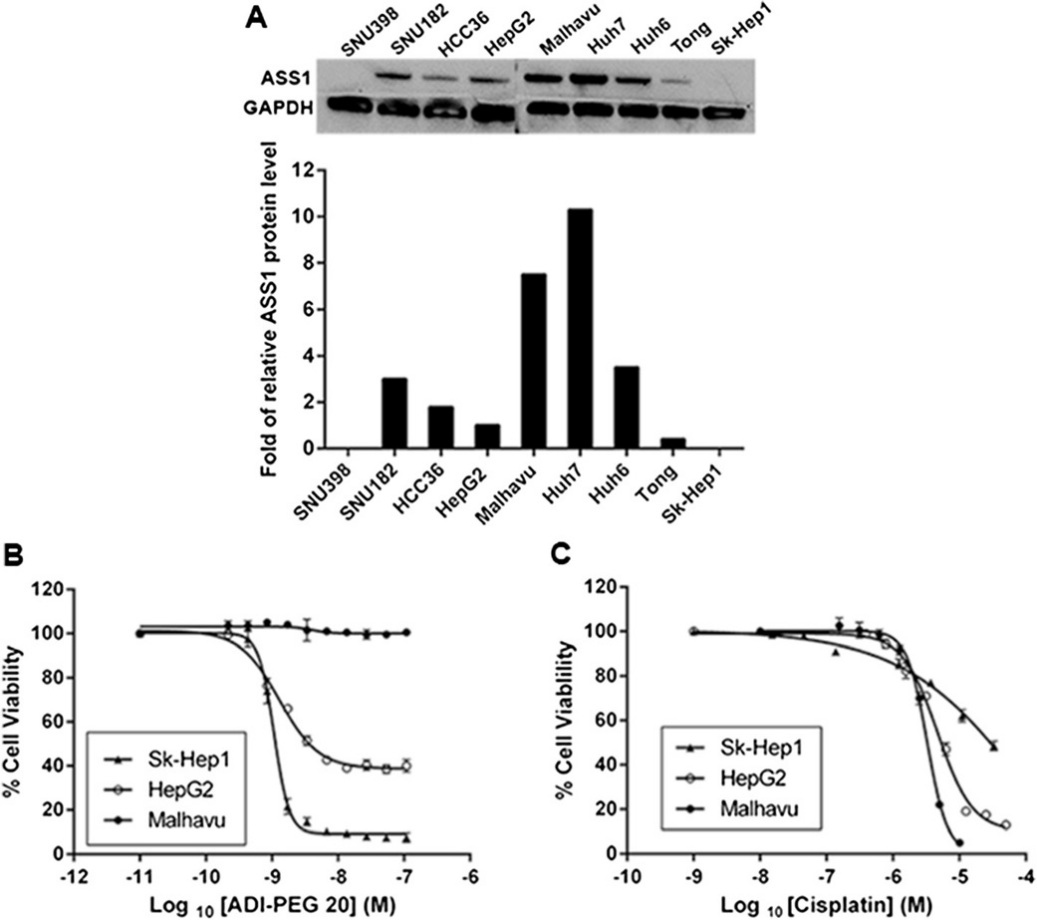
**Effect of ASS1 expression on the sensitivity of HCC cell lines to ADI-PEG 20 and cisplatin treatment. (A)** ASS1 protein levels from cell lysates were determined by western blot. GAPDH was run as a loading control. The signal intensities of the bands were quantified and normalized by taking the level of HepG2 cells as 1. **(B and C)** Sensitivity of three representative HCC cell lines to ADI-PEG 20 and cisplatin. Cells were cultured in medium containing various concentrations of **(B)** ADI-PEG 20 and **(C)** cisplatin. Duplicate samples were assessed for cell viability after 72 h using the Promega luminescence assay. Percent cell viability from either ADI-PEG 20 or cisplatin-treated cells was calculated relative to the viability in corresponding matched DMSO-treated cells, which was designated as 100%. The data are representative of three or more independent experiments. Error bars represent S.D.

To study the relationship between ASS1 expression and sensitivity to ADI-PEG 20 and cisplatin, we first screened 11 human HCC cell lines for this protein. The western blot shown in Figure 1A reveals the different amount of ASS1 protein in select cell lines. The signal intensity of the bands were quantified and normalized by taking the level of ASS1 in the HepG2 cell line as 1. For simplicity, the cell lines were categorized into one of four groups and are designated as either ASS1-high, medium, low or negative (Table 1). ASS1 expression allows cells to utilize citrulline as a substrate for arginine synthesis. ASS1-negative and low lines should thus be sensitive to ADI-PEG 20, while ASS1-high cell lines should be resistant to this drug. As expected, the ASS1-negative and low cell lines Sk-Hep1, SNU398 and Tong are very sensitive to ADI-PEG 20, with IC_50_ values around 1 nM (0.05 μg/mL; Table 1). Cell lines that have some ASS1, designated as ASS1-medium, are less sensitive to ADI-PEG 20, while the ASS1-high cell lines are resistant to this drug. Figure 1B displays ADI-PEG 20 IC_50_ curves for three representative HCC cells lines: Sk-Hep1, HepG2 and Malhavu. As shown, the ADI-PEG 20 curve for the ASS1-negative Sk-Hep1 cell line is very steep, with only 5-10% cell viability remaining at the highest concentration of ADI-PEG 20 (100 nM; 5 μg/mL), while this drug only kills 50% of the ASS1-medium HepG2 cells at the same concentration. In contrast, the ASS1-high line Malhavu is completely resistant to ADI-PEG 20.

**Table 1:** **Sensitivity of human HCC cell lines to ADI-PEG 20 and cisplatin treatment**

Cell line	ASS1 level	ADI-PEG 20 IC _50_(nM)	Cisplatin IC _50_(μM)
Huh7	High	> 10^a^	2.7 ± 0.3
Malhavu	High	No curve	3.3 ± 0.2
Huh6	High	No curve	2.9 ± 0.8
SNU182	High	No curve	1.3 ± 0.3
Hep3B	High	> 10^a^	0.36 ± 0.02
PLC5	High	No curve	2.2 ± 0.3
HCC36	Medium	2.4 ± 0.3	2.9 ± 1.2
HepG2	Medium	1.4 ± 0.2	4.7 ± 0.4
Tong	Low	1.3 ± 0.1	>30^b^
Sk-Hep1	Negative	1.1 ± 0.1	>30^b^
SNU398	Negative	1.2 ± 0.1	23 ± 7.8

HCC cells were treated with various concentrations of either ADI-PEG 20 or cisplatin and the Promega luminescence assay was performed. IC_50_ was calculated on replicates (n = 3 to 4, mean ± SD).

^a^No good IC_50_ fit: approximately 25-30% loss of cell viability by 10 nM ADI-PEG 20.

^b^No good IC_50_ fit: lower limit.

A reciprocal relationship between ASS1 expression and platinum resistance has been previously observed in ovarian cancer [22, 36]. Interestingly, the HCC cell lines also show different sensitivity to cisplatin depending on the level of ASS1 present in each line. All of our ASS1-negative or low cell lines are resistant to cisplatin, with Tong and Sk-Hep1 both having an IC_50_ value above 30 μM (Table 1). The ASS1-high cell lines are at least 5 to 10-fold more sensitive to cisplatin, with IC_50_ values in the low micromolar for most. Figure 1C shows cisplatin IC_50_ curves for three representative HCC cells lines: Sk-Hep1, HepG2 and Malhavu. Surprisingly, this reciprocal trend between ASS1 expression and cisplatin resistance that we observe in our HCC cell lines is not seen with other platinum therapies such as oxaliplatin and carboplatin, or other chemotherapies such as doxorubicin, docetaxel, 5-FU or gemcitabine (data not shown). In addition, cisplatin IC_50_ values have been obtained for multiple cell lines from three other cancer types (colorectal cancer, melanoma and non-small cell lung cancer) and no such trend is observed, at least in the cell lines tested (data not shown). Thus, this reciprocal relationship between ASS1 expression and cisplatin may be exclusive to HCC and ovarian cancer. In summary, our data thus far indicate that ADI-PEG 20 is effective at killing ASS1-negative HCC cells, while cisplatin is effective at killing ASS1-positive HCC cells. This observation suggests that their combination should be an efficacious treatment for heterogeneous cell populations that exist in tumors.

### Methylation status of the ASS1 promoter correlates with sensitivity to ADI-PEG 20 and resistance to cisplatin

**Figure 2:**
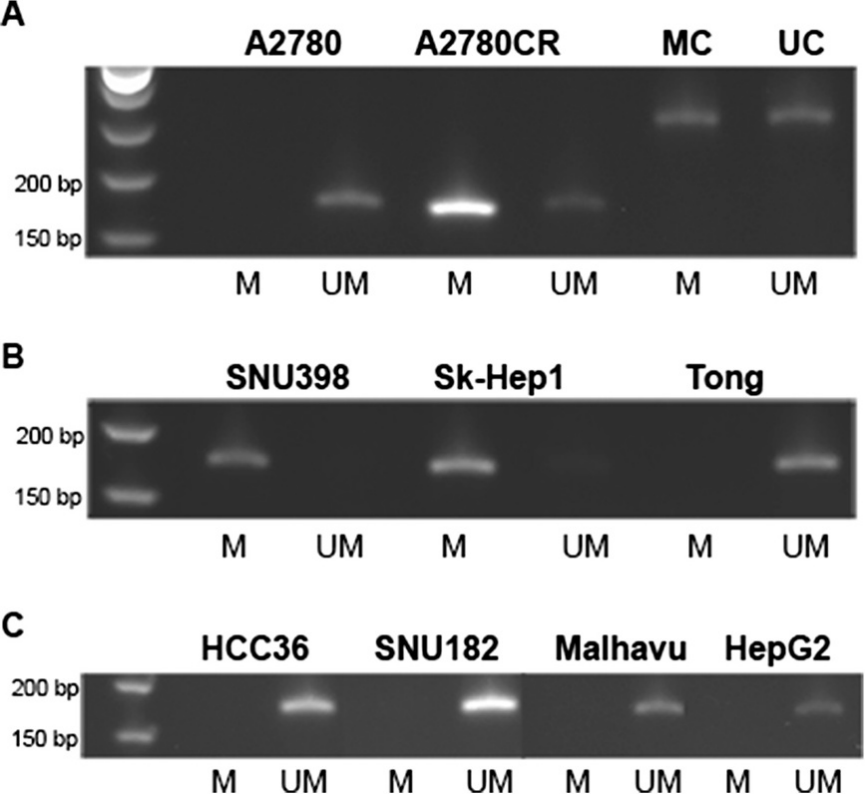
**Methylation status of the ASS1 promoter in HCC cancer cell lines.** DNA bisulfite conversion was performed directly from the cells and MSP was subsequently carried out to determine the methylation status of the ASS1 promoter. UM denotes unmethylated and M denotes methylated. Unmethylated and methylated controls (UC and MC) were included to assess bisulfite conversion efficiency. Expected band sizes are as follows: ASS1: 188 bp; control DNA: 274 bp. Data is representative of 3-4 independent experiments. **(A)** Control: ovarian cell lines A2780 and A2780CR. **(B)** ASS1-negative/low cells: SNU398, Sk-Hep1 and Tong. **(C)** ASS1-positive cells: HCC36, SNU182, Malhavu and HepG2.

Epigenetic silencing via promoter CpG methylation in cell lines lacking ASS1 expression has been demonstrated in multiple cancer types [4, 22–24, 26, 27]. A strong correlation between the methylation of the ASS1 promoter and sensitivity to ADI-PEG 20 has been established in these various cancer cell lines. In addition, an important role for ASS1 in regulating platinum sensitivity via DNA methylation-dependent epigenetic regulation of the ASS1 promoter has been observed in ovarian cancer [22]. This study demonstrated the presence of methylated CpG dinucleotides in the ASS1 promoter of a cisplatin resistant A2780CR cell line, while the parental A2780 line was essentially unmethylated. Using the A2780 and A2780CR ovarian cell lines as controls, we show the ASS1 DNA in the A2780 cell line is entirely unmethylated, while it is almost completely methylated in the A2780CR cell line (Figure 2A). To determine if our ASS1-negative HCC cisplatin-resistant cell lines are epigenetically regulated, we examined the methylation status of the ASS1 promoter in the Sk-Hep1 and SNU398 cell lines. As expected, both of these cisplatin-resistant, ASS1-deficient cell lines are completely methylated at the ASS1 promoter (Figure 2B), confirming that the silencing of ASS1 in these HCC cell lines is indeed epigenetic-based. In addition, Sk-Hep1 and SNU398 are very sensitive to arginine deprivation, demonstrating that methylation of the ASS1 promoter also correlates with sensitivity to ADI-PEG 20 in HCC. Furthermore, all of our ASS1-positive HCC cell lines are unmethylated (Figure 2C), display sensitivity to cisplatin, and are increasingly resistant to ADI-PEG 20. Surprisingly, the ASS1 promoter is entirely unmethylated in the Tong cell line (Figure 2B), even though these cells contain very little ASS1 protein, are resistant to cisplatin and sensitive to ADI-PEG 20 (Table 1 and Figure 1A). Overall, in HCC, we observe a good association between the methylation status of the ASS1 promoter, ADI-PEG 20 sensitivity and cisplatin resistance in six out of the seven cell lines tested.

### Cisplatin treatment down-regulates ASS1 in ASS1-positive HCC cell lines

**Figure 3:**
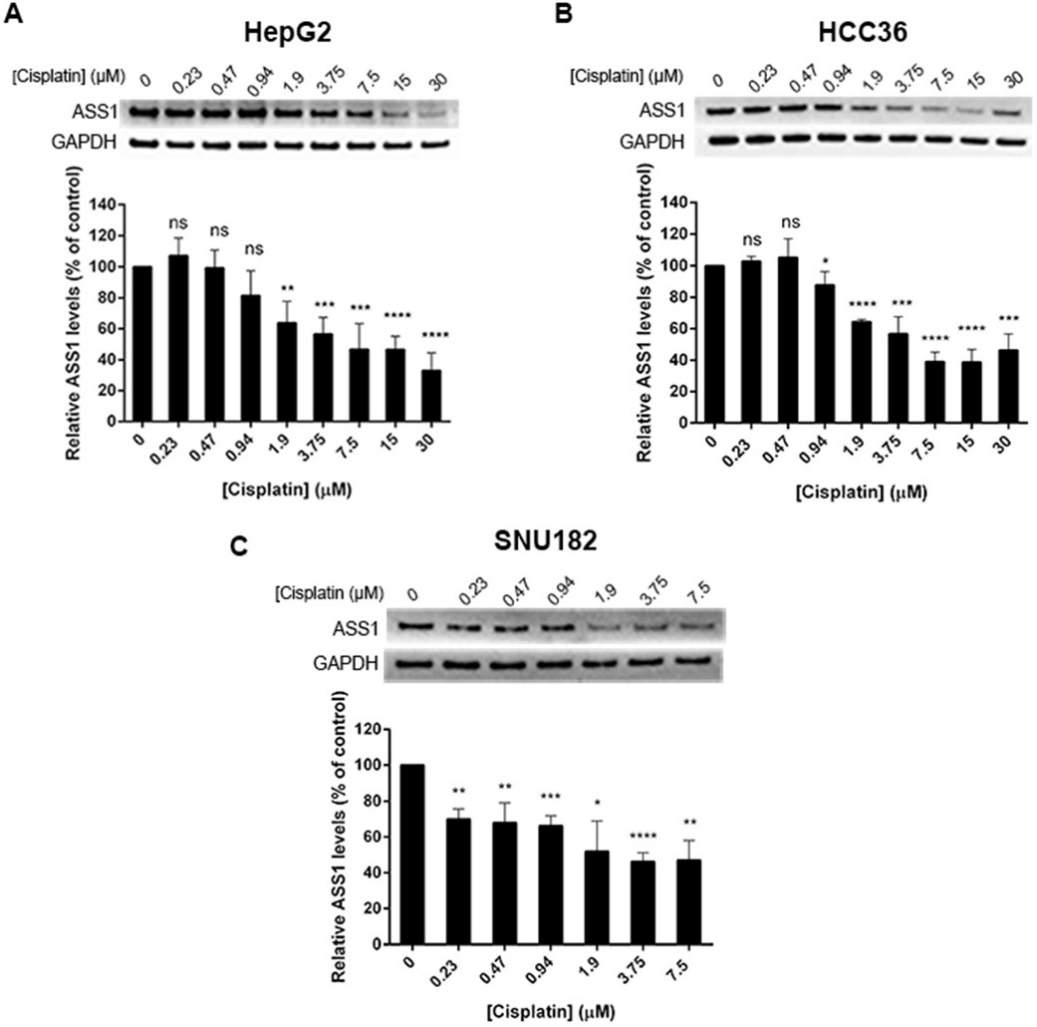
**Cisplatin treatment down-regulates ASS1 protein in HCC cell lines.** Cells were treated with indicated cisplatin concentrations in identical rows of a 96-well microplate. After 72 h, triplicate samples were assessed for cell viability by reading the luminescence and lysates were made out of the remaining identical rows. Luminescence values were used to load equal amounts of protein and ASS1 expression was assessed by western blot. ASS1 levels were normalized to GAPDH at each cisplatin concentration and expressed relative to zero drug (100%). The data are representative of three to four independent experiments. Error bars represent S.D. An unpaired *t*-test was conducted to determine the significance of the change in ASS1 protein levels after each cisplatin concentration treatment as compared to the untreated, or zero drug sample (*p < 0.05, **p < 0.005, ***p < 0.001, ****p < 0.0001). **(A)** HepG2 cells. **(B)** HCC36 cells. **(C)** SNU182 cells.

We have previously demonstrated a reciprocal relationship between ASS1 expression and cisplatin resistance in our HCC cell lines. Therefore, we next investigated whether treatment with cisplatin could down-regulate the ASS1 expression in three representative ASS1-positive HCC cell lines: HepG2, HCC36 and SNU182. Cells were treated in 96-well microplates with increasing cisplatin concentrations for 72 h, and luminescence values, indicative of cell viability, were then used to load similar amounts of protein across all of the cisplatin-treated wells. ASS1 protein expression was subsequently determined by immunoblot analysis and normalized to GAPDH protein at each cisplatin concentration. Figure 3(A and B) shows that ASS1 protein expression is progressively reduced in both the HepG2 and HCC36 cell lines as the cisplatin concentration is increased. The IC_50_ values for cisplatin in the HepG2 and HCC36 cell lines are 4.7 μM and is 2.9 μM, respectively. Thus, the ASS1 levels drop by approximately 50% relative to zero drug at this concentration of cisplatin in the HepG2 cell line and 40% in the HCC36 cells (Figure 3A and B). Further increasing the cisplatin results in even less ASS1 expression, with approximately 70% down-regulation of ASS1 by 30 μM cisplatin in HepG2 cells (Figure 3A). We also studied the ASS1 expression level with cisplatin in the SNU182 cell line, which has the lowest IC_50_ value for cisplatin (1.3 μM) of the three cell lines we chose to investigate. Because cisplatin is more potent in this cell line than the others, there is significant cell death at the higher concentrations of drug, and we found it challenging to use luminescence values to compare the total ASS1 and GAPDH protein at both 15 μM and 30 μM cisplatin with that in the lower concentration wells. For this reason, we have only quantified ASS1 to a concentration of 7.5 μM in the SNU182 cell line (Figure 3C). As with the HepG2 and HCC36 cell lines, we do see a reduction of ASS1 expression in the SNU182 cell line with increasing cisplatin; however, the drop in ASS1 levels is not as gradual. The ASS1 expression decreases by approximately 50% relative to zero drug around the IC_50_ concentration of cisplatin. Taken together, our data indicate that cisplatin treatment reduces ASS1 protein expression in three HCC cell lines. Interestingly, we detect primarily unmethylated DNA at the ASS1 promoter for all three cell lines after the 72 h cisplatin treatment (data not shown), suggesting the mechanism of cisplatin down-regulation during these acute drug treatments is not epigenetic-based.

### ASS1 expression level during ADI-PEG 20 and cisplatin combination treatment is predominantly dictated by ADI-PEG 20

**Figure 4:**
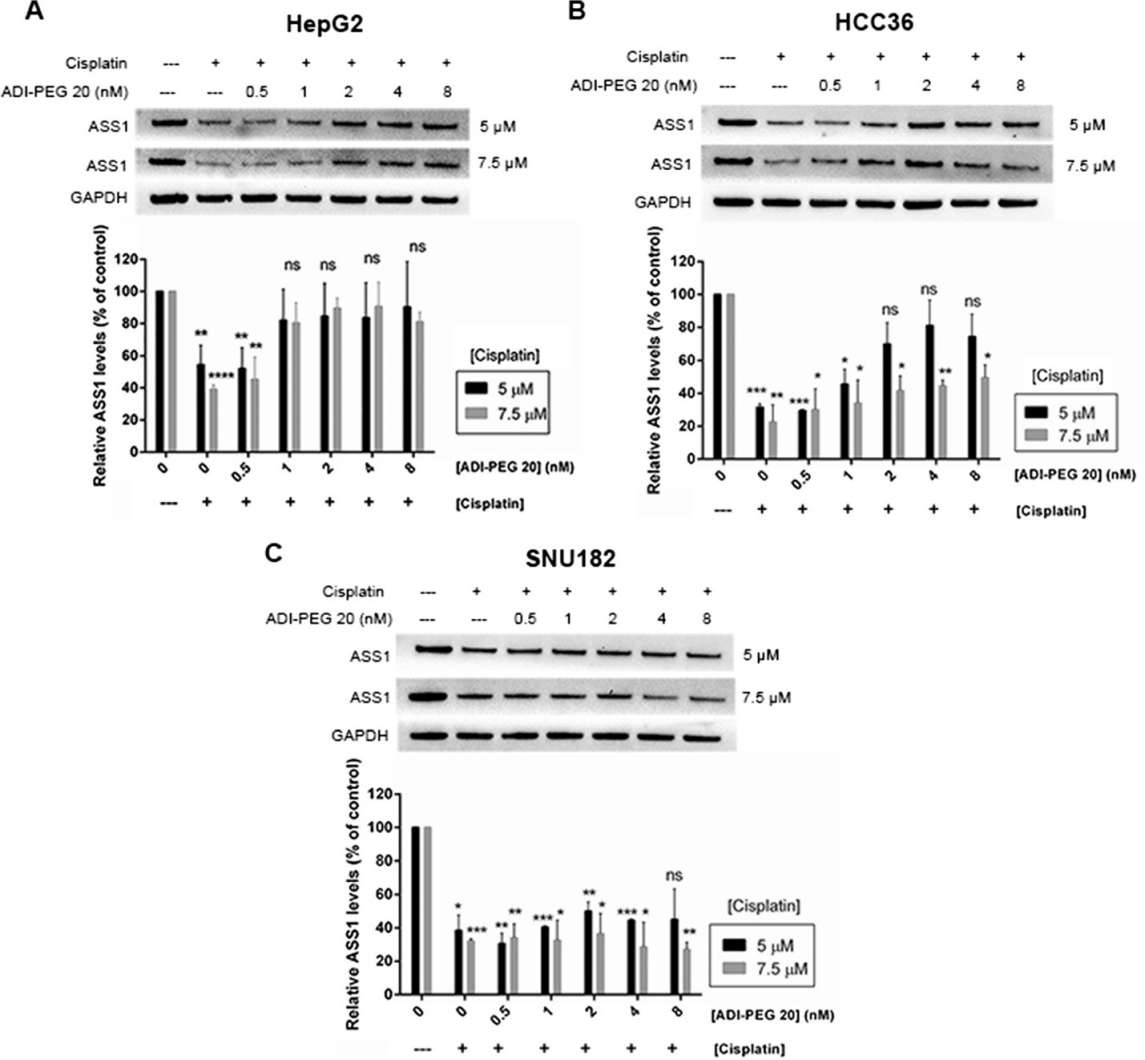
**ASS1 expression during cisplatin and ADI-PEG 20 combination treatment in HCC cells.** Cells were simultaneously treated with a cisplatin concentration of either 5 μM or 7.5 μM and increasing concentrations of ADI-PEG 20: 0.5, 1, 2, 4 and 8 nM in the same well. After 72 h, triplicate samples were assessed for cell viability by reading the luminescence and lysates were made out of the remaining identical rows. Luminescence values were used to load equal amounts of protein and ASS1 levels were assessed by western blot. ASS1 levels were normalized to GAPDH at each treatment condition and expressed relative to zero drug (100%). The data are representative of two to three independent experiments. Error bars represent S.D. An unpaired *t*-test was conducted to determine the significance of the change in ASS1 protein levels after each cisplatin and ADI-PEG 20 combination treatment as compared to the untreated, or zero drug sample (*p < 0.05, **p < 0.005, ***p < 0.001, ****p < 0.0001). **(A)** HepG2 cells. **(B)** HCC36 cells. **(C)** SNU182 cells.

The data thus far demonstrate a correlation between low ASS1 expression, resistance to cisplatin and sensitivity to ADI-PEG 20. Such a relationship provides a situation where one drug drives efficacy in an ASS1-negative cell line (ADI-PEG 20) and the other drives efficacy in an ASS1-high cell line (cisplatin), suggesting a favorable combination drug treatment. Our results demonstrate that cisplatin down-regulates ASS1 in three representative HCC cell lines. Thus, we wanted to determine the effect of the addition of ADI-PEG 20 to cisplatin on the ASS1 levels in these same cell lines. To determine the ASS1 expression during combination drug treatment, cells were simultaneously treated with cisplatin and ADI-PEG 20 in the same wells of a microplate, and then analyzed for ASS1 protein expression. Two concentrations of cisplatin that are known to reduce the ASS1 protein expression were chosen (5 μM and 7.5 μM) and increasing concentrations of ADI-PEG 20 were simultaneously added to the same wells. As expected, for HepG2 cells, treatment at both cisplatin concentrations alone causes a decline in ASS1 protein levels by approximately 50%, which agrees with our previous results (Figure 3A). Figure 4A shows that as ADI-PEG 20 is added to cisplatin at both concentrations, the ASS1 levels increase. At both concentrations of cisplatin and 8 nM ADI-PEG 20 (0.4 μg/mL), the ASS1 expression is almost completely restored to that observed without drug treatment. For the HepG2 cell line, our results indicate that, in combination, ASS1 levels will be dictated by the ADI-PEG 20 at a cisplatin concentration around its IC_50_ value. The same experiment was performed with the HCC36 cell line and we observe a similar trend (Figure 4B). In agreement with previous results, treatment at both 5 μM and 7.5 μM cisplatin alone results in significant down-regulation of ASS1. At both concentrations of cisplatin, ASS1 levels do increase upon addition of ADI-PEG 20, but not to the same extent observed in the HepG2 cell line. We also performed this experiment at 2 μM cisplatin, which is closer to the IC_50_ value for cisplatin in HCC36 cells, and detect a complete return of ASS1 at both 4 and 8 nM ADI-PEG 20 (data not shown). Thus, for the HCC36 cell line, the ASS1 levels will also be determined by the ADI-PEG 20 at a cisplatin concentration around its IC_50_ value.

The last cell line we investigated was SNU182, and surprisingly, we observe a different outcome. At both cisplatin concentrations, the ASS1 levels do not return, even at the highest concentration of ADI-PEG 20 (Figure 4C). The data suggest that in this cell line, ADI-PEG 20 addition is not sufficient to overcome the ASS1 down-regulation induced by cisplatin. One possible explanation is that the SNU182 cell line does have a slightly lower IC_50_ value for cisplatin than the other 2 cell lines (Table 1). Thus, we examined the ASS1 levels in the SNU182 cell line at 2 μM cisplatin, which is closer to its IC_50_ value of 1.3 μM. At 2 μM cisplatin alone, we see 50% down-regulation of ASS1. Interestingly, as ADI-PEG 20 is added, we do not observe any increase in ASS1 expression (data not shown), indicating that cisplatin will dictate ASS1 levels.

## Discussion

The future for the treatment of arginine auxotrophic cancers lies in combination therapies. Several ADI-PEG 20 and cisplatin combination trials are planned. Therefore, understanding the correlation between ASS1 expression and cisplatin and ADI-PEG 20 sensitivities, as well as how ASS1 is regulated by both drugs could provide valuable information for trial design. For the first time, we have shown that there is a reciprocal relationship between ASS1 expression and cisplatin resistance in several human HCC cell lines. We have observed that resistance is specific to cisplatin, as sensitivity to other platinums and chemotherapeutic agents are unaffected by ASS1 expression. In addition, methylation of the ASS1 promoter does associate with sensitivity to ADI-PEG 20, and in HCC, also corresponds with cisplatin resistance, as previously demonstrated in ovarian cancer [22]. These findings suggest that the methylation status of the ASS1 promoter in tumors may predict sensitivity to arginine deprivation with ADI-PEG 20 and also support the future prospect of using methylation profiling to identify which HCC patients may benefit from either cisplatin or ADI-PEG 20.

Our novel data also indicate that cisplatin down-regulates ASS1 protein expression in three HCC cell lines. How exactly cisplatin is affecting ASS1 levels during these acute treatments is currently unknown. Previously published studies indicate that ASS1 regulation occurs at the transcriptional level [37–40]. For example, it has been demonstrated that glutamine stimulated ASS1 expression in Caco-2 cells through O-glycosylation of the transcription factor Sp1 [40], while expression of the ASS1 gene has been shown to be stimulated by interleukin-1β in Caco-2 cells through activation of the transcription factor nuclear factor-ĸβ [38]. In melanoma cells, hypoxia-inducible factor (HIF-1α)-mediated transcriptional repression of ASS1 has been observed [31, 33]. Other factors have been shown to positively or negatively regulate ASS1 expression. For example, cAMP increases ASS1 expression, while fatty acids cause suppression of this protein [41, 42], and factors such as hormones and pro-inflammatory stimuli are also known to regulate ASS1 expression [39, 43].

Interestingly, there is suggestion that acquired resistance to cytotoxic agents occurs predominantly via epigenetic events [44, 45]. A significant function for ASS1 in regulating platinum sensitivity via methylation of the ASS1 promoter has been observed in ovarian cancer utilizing the A2780 and A2780CR cell lines [22]. The A2780CR cell line was established by intermittent exposure of the parental A2780 cell line to stepwise, increasing concentrations of cisplatin up to a concentration of 8 μM over a period of approximately 9 months [46]. This cell line was found to be 7.3-fold more resistant than the parental line, and it was indicated that this degree of resistance in the A2780CR cell line was stable for at least nine months during subculture in drug-free medium. Our experience with a commercially available A2780CR cell line is similar. We have observed that A2780CR does not express ASS1, is 12-fold more resistant to cisplatin than the parental cell line, and is completely methylated at the ASS1 promoter after being subcultured in cisplatin-free medium for 2 months. Given the similarities to ovarian cancer that we have observed in our HCC cell lines regarding ASS1 expression, methylation status of the ASS1 promoter and cisplatin resistance, we are currently establishing a HepG2 cisplatin-resistant (HepG2CR) cell line by progressively exposing HepG2 cells to increasing cisplatin. Preliminary data indicate a three-fold IC_50_ value increase for cisplatin in HepG2CR over the parental cell line after only one month of drug exposure. Once we acquire a more permanent resistant phenotype, we will determine the methylation status of HepG2CR and perform other analyses to understand the mechanisms of acquired cisplatin resistance in HCC.

Several ADI-PEG 20 combination trials are ongoing or planned, including a combination with cisplatin for metastatic melanoma, ovarian cancer and other solid tumors, docetaxel for prostate and non-small cell lung cancer (NSCLC), doxorubicin for breast cancer, and cisplatin and pemetrexed for NSCLC and malignant pleural mesothelioma [20]. We have determined that ASS1 loss is a biomarker of cisplatin resistance and ADI-PEG 20 sensitivity, whereas ASS1 positivity is an indicator of cisplatin sensitivity and ADI-PEG 20 resistance in HCC cell lines. This observation suggests that a cisplatin and ADI-PEG 20 regimen should be superior to either drug alone for the treatment of HCC patients. To examine the potential for the use of ASS1 as a predictor in combination therapy, we sought to determine the ASS1 levels in HCC cells with both drugs present. Predictably, we found that the ASS1 protein levels will be dictated by one of the two drugs and is concentration-and cell-line dependent. In two of the three cell lines tested, the ASS1 levels seemed to be controlled by ADI-PEG 20, while cisplatin was able to maintain low ASS1 levels in the remaining cell line. Obviously, it is hard to predict clinical behavior from cell-based assays. We believe that ADI-PEG 20 will influence the ASS1 level of this two-drug regimen at more clinically relevant concentrations, resulting in higher ASS1 levels. This observation suggests that long term treatment with this combination could result in cisplatin resistant cells becoming cisplatin sensitive. Furthermore, several groups have observed that reduced expression of ASS1 is significantly associated with advanced tumor stage and an association with a worse clinical outcome [21–25]. These observations imply that the higher ASS1 levels present with the addition of ADI-PEG 20 to cisplatin may elicit better clinical outcomes for HCC patients. Extending these observations further to the clinic, our results suggest that while ASS1 may be a predictive biomarker for either ADI-PEG 20 or cisplatin as a single agent or pre-therapy, the status of this indicator may change by addition of the second drug and possibly evolve during tumor progression or metastasis. This concept of intratumoral heterogeneity within the same patient is growing in recognition and discordance of predictive or prognostic biomarker testing results between primary tumor and metastases or resistance acquisition has been reported in several tumor types [47].

## Conclusion

Our data support the rationale of combining cisplatin and ADI-PEG 20 in the clinical treatment of HCC. We believe these two drugs will be complementary in a clinically relevant heterogeneous tumor. Furthermore, in HCC, sensitivity to ADI-PEG 20 may be superior in cases that have relapsed after cisplatin-based chemotherapy. Extending beyond HCC to other cancers, our results suggest that in the combination setting, a patient does not necessarily need to have an ASS1-deficient tumor to reap benefit from an ADI-PEG 20 and cisplatin drug treatment.

## Abbreviations

ASS1: Argininosuccinate synthetase
HCC: Hepatocellular carcinoma
ADI-PEG 20: PEGylated arginine deiminase
IC_50_: Half maximal inhibitory concentration
GAPDH: Glyceraldehyde 3-phosphate dehydrogenase
MSP: Methylation-specific PCR
TSS: Transcription start site
HIF-1α: Hypoxia-inducible factor
HepG2CR: HepG2 cisplatin-resistant cell line
NSCLC: Non-small cell lung cancer.
